# Discovering Genotype Variants in an Infant with VACTERL through Clinical Exome Sequencing: A Support for Personalized Risk Assessment and Disease Prevention

**DOI:** 10.3390/pediatric13010006

**Published:** 2021-01-05

**Authors:** Gloria Pelizzo, Luigi Chiricosta, Emanuela Mazzon, Gian Vincenzo Zuccotti, Maria Antonietta Avanzini, Stefania Croce, Mario Lima, Placido Bramanti, Valeria Calcaterra

**Affiliations:** 1 Pediatric Surgery Unit, Ospedale dei Bambini “Vittore Buzzi”, 20154 Milano, Italy; 2 Department of Biomedical and Clinical Science “L. Sacco”, University of Milano, 20157 Milano, Italy; gianvincenzo.zuccotti@unimi.it; 3 IRCCS Centro Neurolesi “Bonino-Pulejo”, 98124 Messina, Italy; luigi.chiricosta@irccsme.it (L.C.); emanuela.mazzon@irccsme.it (E.M.); placido.bramanti@irccsme.it (P.B.); 4 Department of Pediatrics, Ospedale dei Bambini “Vittore Buzzi”, 20154 Milano, Italy; valeria.calcaterra@unipv.it; 5 Immunology and Transplantation Laboratory, Cell Factory, Pediatric Hematology Oncology Unit, Department of Maternal and Children’s Health, Fondazione IRCCS Policlinico S. Matteo, 27100 Pavia, Italy; ma.avanzini@smatteo.pv.it (M.A.A.); stefania_croce186@yahoo.it (S.C.); 6 Pediatric Surgery Unit, S. Orsola Hospital, University of Bologna, 40138 Bologna, Italy; mario.lima@unibo.it; 7 Pediatrics and Adolescentology Unit, Department of Internal Medicine, University of Pavia, 27100 Pavia, Italy

**Keywords:** VACTERL, exome sequencing, infant, healthcare, personalized, prevention, risk

## Abstract

Congenital anomalies may have an increased risk of noncommunicable diseases (NCDs) We performed a clinical exome analysis in an infant affected by “Vertebral, Anorectal, Cardiac, Tracheoesophageal, Genitourinary, and Limb” (VACTERL) malformation association to identify potential biomarkers that may be helpful for preventing malignancy risk or other chronic processes. Among the variants, six variants that may be linked with VACTERL were identified in the exome analysis. The variants c.501G>C on *OLR1* and c.-8C>G on *PSMA6* were previously associated with myocardial infarction. The variants c.1936A>G on *AKAP10* and c.575A>G on *PON1* are linked to defects in cardiac conduction and artery disease, respectively. Alterations in metabolism were also suggested by the variants c.860G>A on *EPHX2* and c.214C>A on *GHRL*. In addition, three variants associated with colon cancer were discovered. Specifically, the reported variants were c.723G>A on *CCND1* and c.91T>A on *AURKA* proto-oncogenes as well as c.827A>C in the tumor suppressor *PTPRJ*. A further inspection identified 15 rare variants carried by cancer genes. Specifically, these mutations are located on five tumor suppressors (*SDHA*, *RB1CC1*, *PTCH1*, *DMBT1*, *BCR*) and eight proto-oncogenes (*MERTK*, *CSF1R*, *MYB*, *ROS1*, *PCM1*, *FGFR2*, *MYH11*, *BRCC3*) and have an allele frequency lower than 0.01 in the Genome Aggregation Database (GnomAD). We observed that the cardiac and metabolic phenotypic traits are linked with the genotype of the patient. In addition, the risk of developing neoplasia cannot be excluded a priori. Long-term surgical issues of patients with VATER syndrome could benefit from the clinical exome sequencing of a personalized risk assessment for the appearance of further disease in pubertal timing and adult age.

## 1. Introduction

“Vertebral, Anorectal, Cardiac, Tracheoesophageal, Genitourinary, and Limb” (VACTERL/VATER) malformation association is a rare condition characterized by the presence of at least three of the following congenital malformations: vertebral defects, anal atresia, cardiac defects, tracheoesophageal fistula, renal anomalies, and limb abnormalities [1,2]. Other congenital anomalies (Cas) may be also present, including airway malformations. No major genetic risk factors are known to be involved in the etiology of VACTERL, and multifactorial pathogenesis has been proposed [1,2]. The management of patients with VACTERL association can be complex and can result in longer-term sequelae [1,2].

Cas represent one of the main causes of fetal death, infant mortality and morbidity, and long-term complications. Although the pathogenesis of Cas is still unknown, complex interactions between genes and the environment have been proposed. This multifactorial interaction modifies the normal embryo-fetal development, especially during the organogenesis phase [3]. The organism will retain memories of the insult immediately, and the adaptive response may result in pathology later on [4,5]. In particular, an increased risk of noncommunicable diseases (NCDs), including type 2 diabetes, cardiovascular disorders, and cancer, is also reported during childhood [4,5]. In patients with multiple congenital malformations, such as VACTERL, the risk of NDCs in pediatric and adult age is not defined but could be considered.

Given the recent advances in scRNAseq, its application in human diseases may enable a better understanding of pathological processes [4]. The identification of the gene expression patterns may be useful to predict the risk of developing chronic diseases and personalizing their prevention and treatment [4,5,6,7,8].

We performed a clinical exome analysis in an infant affected by the VACTERL association to identify potential biomarkers helpful for early detection of risk malignancy and chronic degenerative processes. A potential personalized prevention strategy could also allow personalized treatment.

## 2. Case Report

The patient was a six-month-old girl, born to Chinese nonconsanguineous parents. Diabetes and exposure to other environmental factors during pregnancy were not recorded. No previous pregnancies with congenital malformations were noted, and family history was also unremarkable. She was diagnosed with VACTERL association due to the presence of an imperforated anus with rectovestibular fistula, sacral vertebral anomalies and coccygeal agenesis, vesicoureteral reflux, and complex cardiovascular anomalies (double-outlet right ventricle and subaortic stenosis associated with ventricular septal defect). Cardiosurgery was performed at the age of four months. At six months, the baby was readmitted for treatment of the anorectal anomaly. Before general anesthesia induction for anorectal malformation repair, a severe and rare long-segment congenital tracheal stenosis was detected, and a slide tracheoplasty was subsequently performed. The postoperative course was uneventful.

The parents of the patient provided informed consent for genetic testing and publication.

**Figure 1:**
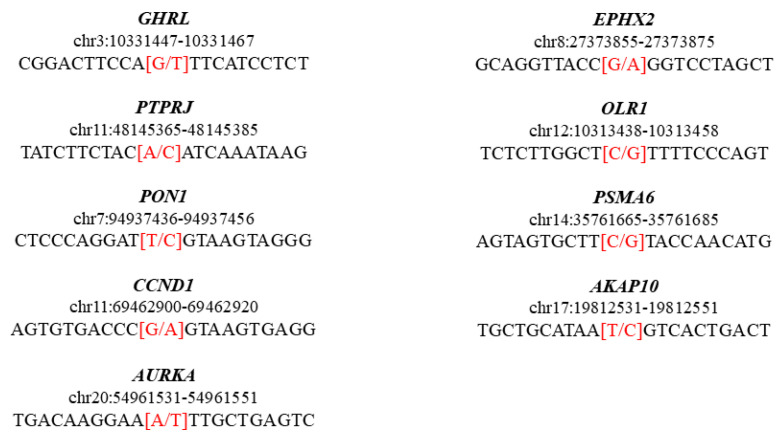
Genomic sequence representation of the variants identified as pathogenic or a risk factor in the clinical exome. The bases from position-10 to position+10 are represented for each variant, and the genomic changes are highlighted in red.

We conducted an analysis of the clinical exome of the patient in triplicate. The gene panel TruSight One was retrieved by Illumina and sequenced on the MiSeq platform as paired-ends using reads 150 nucleotides in length. The final variant call format (VCF) included 4240 variants. All of the found variants passed all the filters. To be included in the final VCF, each variant had to be called at least two out of three times in the analysis. All the variants were associated with the up-to-date version of dbSNP (151) and ClinVar (at 16/07/2020). Among them, we found two heterozygous variants described as pathogenic (rs1566734, rs72474224), one heterozygous pathogenic/risk factor (rs696217), three homozygous (rs662, rs9344, rs2273535), and four heterozygous risk factors (rs751141, rs11053646, rs1048990, rs203462) (Table 1). We highlighted the sequence window of the variants in Figure 1.

**Figure 2:**
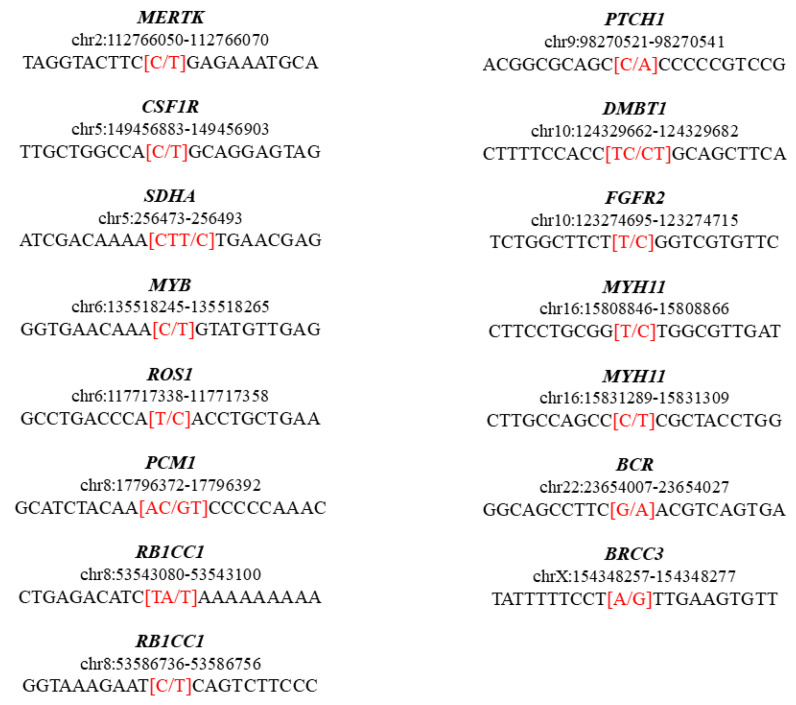
Genomic sequence representation of the rare variants identified as tumor suppressors or proto-oncogenes in the clinical exome. The bases from position-10 to position+10 are represented for each variant, and the genomic changes are highlighted in red.

Moreover, 2279 variants were associated with no clinical phenotype, and no variant was associated with VACTERL disease. In order to investigate the analysis in depth, we used the keyword “VACTERL” (Disease/Phenotype) on ClinVar to retrieve all the genes associated with the disease. The result showed 98 items on 8 genes (*BAZ1A*, *FANCB*, *FANCL*, *HOXD13*, *PTEN*, *KLLN*, *SALL1*, *ZIC3*). The only variant in our analysis affecting these genes is rs4614723, carried out by *SALL1* but defined as benign. Given the predisposition of the patients suffering from VACTERL to develop pancreatic and esophageal cancers, we also inspected the genes associated with the keyword “pancreatic cancer” (Disease/Phenotype) and “esophageal cancer” (Disease/Phenotype) in ClinVar. The queries returned 250 items on six genes for pancreatic cancer and one item on a gene for esophageal cancer. The only genes having variants associated with the patient were *BRCA2* and *PALLD* for pancreatic cancer, but they are clinically identified as benign. Furthermore, we extracted a list of proto-oncogenes and tumor suppressor genes from UniProtKB using the filters “Human” and “Reviewed” and selected only the variants carried on these genes. We then enriched the VCF file using ANNOVAR [9] to obtain its frequency for each variant. We identified nine rare variants on eight proto-oncogenes (*MERTK*, *CSF1R*, *MYB*, *ROS1*, *PCM1*, *FGFR2*, *MYH11*, *BRCC3*) and six rare variants on five tumor suppressors (*SDHA*, *RB1CC1*, *PTCH1*, *DMBT1*, *BCR*), Figure 2. Specifically, the allele frequency of these rare variants is lower than 0.01 in the Genome Aggregation Database (GnomAD) [10] and not reported in ClinVar as benign in the supported publications (Table 2). None of these genes were already associated with VACTERL in the literature.

## 3. Discussion

Structural birth defects occur in approximately 3% to 6% of all live births [11]. Most structural birth defects develop early in embryogenesis during the first 10 weeks of pregnancy [11], and the vast majority of birth defects are “nonchromosomal anomalies” characterized, as in VACTERL association, by multiorgan involvement. The mechanisms by which environmental or genetic insults disrupt fetal development are not fully understood. However, there is no doubt that an adverse environment in utero leads to permanent changes in the structure of organs and systems, which have a key role in “fetal programming.” Fetal adaptation is responsible for an increased risk of NCDs and other chronic diseases, such as obesity, which, itself, is a major risk factor for NCDs throughout the life-course [3,5].

In this sense, NGS analysis may be useful to discover genetic aberration and support clinicians, providing a link to the disease.

The clinical exome analysis of infants with VACTERL performed in our study highlighted several genetic variants (Figure 1).

The polymorphism c.501G>C on *OLR1* gene encodes for oxidized low-density lipoprotein receptor 1, and the c.-8c>G on *PSMA6* gene encodes for proteasome subunit alpha type-6. These two heterozygous variants are associated with myocardial infarction in ClinVar. Specifically, c.501G>C is a missense variant that causes a change in the protein sequence of oxidized low-density lipoprotein receptor 1 and may result in reduced interaction with ligands. Tatsuguchi et al. observed a group of patients suffering from myocardial infarction and suggested that this variant promotes atherogenesis and coronary artery disease [12]. On the other hand, several studies were performed on the 5′ UTR variant c.-8c>G, leading to a premature start.

In a Japanese population, Ozaki et al. found a significant association of this variant with myocardial infarction pathogenesis via the activation of inflammatory processes [13], and Hinoara et al. proposed a modest risk factor of the variant in coronary artery disease [14].

In addition, the heterozygous variant c.1936A>G on the *AKAP10* gene, which encodes for A-kinase anchoring protein 10, is associated with susceptibility to cardiac conduction defect, and the homozygous variant c.575A>G on *PON1*, which encodes for serum paraoxonase and arylesterase 1, is reported with susceptibility to coronary artery spasm and artery disease.

Krammerer et al. found a strong correlation with c.1936A>G and aging, which, interestingly, seems to alter heart functionality. Indeed, the PR interval of the electrocardiogram is reduced in subjects that carry the variant. In an in vitro experiment, the authors observed a change in the ability of A-kinase anchoring protein 10 to bind the isoform of protein kinase A (PKA-Riα), altering the signal mediated by cAMP [15]. Ito et al. suggested that oxidative stress may be not properly suppressed when c.575A>G polymorphism occurs, facilitating the genesis of coronary spasm [16].

In line with these considerations, it is interesting to observe the heterozygous variants c.860G>A on *EPHX2* and c.214C>A on *GHRL* genes that expose the patients of this study to metabolic syndrome and familial hypercholesterolemia 1, respectively. Specifically, *EPHX2* encodes for the soluble epoxidase hydrolase with lipid-phosphate phosphatase activity that regulates cardiovascular functions. Interestingly, Fornage et al. found a twofold greater risk of developing coronary artery calcification in young people carrying c.860G>A [17]. Moreover, Othoshi et al. suggested that this variant may lead to insulin resistance in the pathogenesis of type 2 diabetes [18]. *GHRL* encodes for the hormone ghrelin, which prepares food intake by the secretion of gastric acid and increases gastric motility [19]. Ghrelin regulates energy homeostasis, and Imaizumi et al. proposed this variant as a risk factor for obesity, leading to an increase in body mass index [20].

Childhood cancer risk in chromosomal anomalies has been described well, such as acute lymphoblastic leukemia in children with Down syndrome [21,22] or retinoblastoma in patients with chromosome 13q14 deletion syndrome [23]. More recently, Norwood et al. reported that any congenital anomaly, including nonchromosomal anomalies, was associated with an increased risk of cancer for several cancer types, including neuroblastoma, renal, hepatoblastoma, soft-tissue sarcoma, and germ cell tumors, during childhood [3].

The pathogenic mechanism of the link between congenital anomalies and cancer remains to be elucidated. Plausible theories include environmental exposures leading to both conditions, somatic mutations in developmental genes early in embryogenesis or overexpressed genes, or altered pathways, including both developmental and cancer predisposition genes [3].

Our analysis highlights the heterozygous variant c.827A>C on the tumor suppressor gene *PTPRJ* and the homozygous variants c.723G>A and c.91T>A on the proto-oncogenes *CCND1* and *AURKA*, respectively. These variants are associated with colon cancer in ClinVar. Specifically, *PTPRJ* encodes for the receptor-type tyrosine-protein phosphatase eta, an enzyme that regulates angiogenesis, cell growth, proliferation, differentiation, and migration. Mita et al. observed a highly increased risk of developing colon cancer when c.827A>C is present simultaneously with p.Arg326Gln on the same gene [24]. Interestingly, the patient carries both variants.

Hryhomorowicz et al. studied c.723G>A and identified it as a risk factor for thyroid carcinoma even if with low penetrance, especially when it is in homozygosity, as in our study [25]. In a comparative study, Weinhold et al. found a significant association between this variant and the translocation of a portion of chromosome 11, resulting in a risk factor for multiple myeloma [26]. Moreover, Absenger et al. inspected the prognostic potential of the variant, suggesting it as a possible biomarker in colon cancer [27]. Ewart-Toland et al. studied the c.91T>A substitution in rat models, discovering an increased association of the variant with aneuploidy in human colon tumors. Indeed, the variant strengthens the binding of aurora kinase A with the E2 ubiquitin-conjugating enzyme, altering the cell cycle progression [28].

In addition, we analyzed the tumor suppressors or proto-oncogenes *BCR*, *BRCC3*, *CSF1R*, *DMBT1*, *FGFR2*, *MERTK*, *MYB*, *MYH11*, *PCM1*, *PTCH1*, *RB1CC1*, *ROS1*, and *SDHA,* which carried very rare variants in our analysis (Figure 2). They have never been directly associated with VACTERL.

In detail, one heterozygous missense variant was identified on the *BCR* gene encoding for a guanine nucleotide exchange factor, whose activity is identified as a serine/threonine kinase [29]. When *BCR* genes fuse with the translocated *ABL1* gene, they cause uncontrolled cell division in chronic myeloid leukemia [30]. Another heterozygous splice region variant is identified on the gene *BRCC3* that encodes for the Lys-63-specific deubiquitinase BRCC36 protein, a subunit of the BRCA1-BRCA2-containing complex. It is involved in the DNA damage response and was associated with myeloid neoplasms [31,32]. *CSF1R* carries one heterozygous missense variant. *CSF1R* encodes for the tyrosine kinase transmembrane macrophage colony-stimulating factor 1 receptor. *CSF1R* regulates the survival, proliferation, and differentiation of macrophages, along with monocytes interacting with CSF1 and IL34 [33] and actin cytoskeleton reorganization, cell migration, and cancer cell invasion through the ERK1/2 and JNK pathways [34]. It is associated with pediatric-onset leukoencephalopathy and brain malformation when absent in the brain [35,36]. In addition, one homozygous splice region variant of two close nucleotides was identified on the *DMBT1* gene. The *DMBT1* gene encodes for deleted in malignant brain tumors 1. It is a candidate tumor suppressor gene for colorectal, gastric, esophageal, lung, and brain cancers [37,38,39,40], probably influencing the immune system [41]. One heterozygous missense variant was discovered on the *FGFR2* gene. *FGFR2* encodes for fibroblast growth factor receptor 2, a tyrosine-protein kinase that regulates cell proliferation, differentiation, and apoptosis specifically during embryonic development [42,43,44], controlling lung morphogenesis and skeleton and skin development [45]. *MERTK* includes one heterozygous splice region variant. *MERTK* encodes for the tyrosine-protein kinase Mer, a member of the TAM receptor tyrosine kinases involved in cytokine release, cell proliferation, and migration. Mutations on *MERTK* are associated with autoimmune diseases, and expression alterations may have oncogenic potential [46]. The transcriptional activator Myb is encoded by *MYB,* which had a heterozygous missense variant in our analysis. It is a transcription factor mainly involved in the proliferation and differentiation of hematopoietic cells and plays an important role in breast and salivary adenoid cystic carcinoma [47,48]. *MYH11* has one missense heterozygous and one heterozygous splice region variant. It encodes for Myosin-11 and is mainly involved in muscle contraction; however, when altered, it may contribute to intestinal [49], gastric, and colorectal [50] cancers and acute myeloid leukemia [51]. *PCM1* carries a heterozygous missense mutation of two close nucleotides. It encodes for the pericentriolar material 1 protein, required for the assembly and functioning of the centrosome and to attach microtubules [52], and is involved in the chromosomal rearrangement in myeloid or lymphoid neoplasms [53,54]. *PTCH1* has one homozygous missense variant. *PTCH1* encodes for protein patched homolog 1, essential in embryogenesis [55]. Mutations of this gene have already been associated with holoprosencephaly [56] along with several cancers such as nevoid basal cell carcinoma syndrome and medulloblastoma [57,58]. *RB1CC1* carries two heterozygous variants, one missense in the coding sequence and one deletion in an intron and splice region. This gene encodes for RB1-inducible coiled-coil protein 1, which plays a key role in the initiation of autophagy, the impairment of which increases cell death [59,60]. This protein also acts as a transcription factor for retinoblastoma 1 and seems to regulate the progression of various cancers [61]. *ROS1* carries a heterozygous splice region variant. This gene encodes for the proto-oncogene tyrosine-protein kinase ROS, which is an integral membrane protein receptor that functions as a growth and differentiation factor via PI3K-mTOR, STAT3, or VAV3 signaling [62,63]. Its rearrangement is associated with lung cancer, glioblastoma, ovarian carcinoma, sarcoma, and cholangiocarcinoma [64,65]. *SDHA* has a heterozygous frameshift variant. It encodes for the succinate dehydrogenase flavoprotein subunit mitochondrial that in the inner membrane of mitochondria is involved in mitochondrial electron transport chain but seems to represent a link with hereditary tumors [66].

In VACTERL syndrome, clinical exome sequencing could be included as a transformative test for prenatal diagnosis. The prenatal multidisciplinary team approach could benefit from the more accurate detection of a large spectrum of dysmorphologies, described as being part of this complex malformation.

Early detection of the different aspects of the syndrome allows taking charge perinatally, with a potential improvement of the clinical outcome for the child. In addition, most of them, such as tracheal malformations or urogenital malformations, are prenatally unexpected and undetected.

Data from our report could be included in the fetal exome database for the completion of a broad diagnostic capability in pregnancy, with unexpected anomalies. Additionally, considering the possibility for the patient to develop any additional long-term sequelae, a specialist multidisciplinary team for strict clinical monitoring is recommended during childhood and adolescence. Due to the relationship between pubertal timing, growth, and adult health, auxological evaluations should be recommended at least twice a year. Starting from puberty, metabolic profile and cardiologic evaluation may also be annually useful for the early detection of cardio-metabolic risk factors, such as insulin resistance, hypertension, and dyslipidemia.

The current challenge for the future is to translate these approaches into clinical use for surveilling the development of different diseases.

## 4. Conclusions

VACTERL disease is associated with multiorgan impairment, but its etiology is still unclear. In this work, we discovered several variants in a six-month-old patient that could be responsible for the clinical complication of complex malformation. Six of them are related to cardiac dysfunction. c.501G>C (*OLR1*) and c.-8C>G (*PSMA6*) are specifically associated with myocardial infarction, c.1936A>G (*AKAP10*) with cardiac conduction defects, c.575A>G (*PON1*) with artery disease, and c.860G>A (*EPHX2*) and c.214C>A (*GHRL*) with metabolic syndrome. In addition, the proto-oncogenes *CCND1* and *AURKA*, along with the tumor suppressor *PTPRJ*, carry the variants c.723G>A, c.91T>A, and c.827A>C, respectively, which are related to colon cancer. For the first time, we associated nine rare variants on eight proto-oncogenes (*MERTK*, *CSF1R*, *MYB*, *ROS1*, *PCM1*, *FGFR2*, *MYH11*, *BRCC3*) and six rare variants on five tumor suppressor (*SDHA*, *RB1CC1*, *PTCH1*, *DMBT1*, *BCR*) with VACTERL. Clinical exome sequencing could offer support for clinicians to combine the surgical treatment of VACTERL syndrome with a dedicated risk assessment for the prevention of further disease during adolescence and adult age.

## Figures and Tables

**Table 1:** Pathogenic and risk factor variants included in the clinical exome of the patient.

Chromosome	Gene	Position	Rs ID	Reference	Alternative	Variant Type	Consequence	HGVS.c	HGVS.p	Condition	Clinical Significance
chr3	*GHRL*	10331457	rs696217	G	T	SNP HET	Missense variant	c.214C>A	p.Leu72Met	Metabolic syndrome, susceptibility to obesity, age at onset of	Pathogenic, risk factor
chr11	*PTPRJ*	48145375	rs1566734	A	C	SNP HET	Missense variant	c.827A>C	p.Gln276Pro	Carcinoma of colon	Pathogenic
chr7	*PON1*	94937446	rs662	T	C	SNP HOM	Missense variant	c.575A>G	p.Gln192Arg	Enzyme activity finding coronary artery disease, susceptibility to|Coronary artery spasm 2, susceptibility to	Risk factor
chr11	*CCND1*	69462910	rs9344	G	A	SNP HOM	Splice region variant and synonymous variant	c.723G>A	p.Pro241Pro	Von Hippel–Lindau syndrome, modifier of colorectal cancer, susceptibility to multiple myeloma, translocation 11,14 type	Risk factor
chr20	*AURKA*	54961541	rs2273535	A	T	SNP HOM	Missense variant	c.91T>A	p.Phe31Ile	Colon cancer, susceptibility to	Risk factor
chr8	*EPHX2*	27373865	rs751141	G	A	SNP HET	Missense variant	c.860G>A	p.Arg287Gln	Familial hypercholesterolemia 1	Risk factor
chr12	*OLR1*	10313448	rs11053646	C	G	SNP HET	Missense variant	c.501G>C	p.Lys167Asn	Myocardial infarction	Risk factor
chr14	*PSMA6*	35761675	rs1048990	C	G	SNP HET	5 prime UTR premature start codon gain variant	c.-8C>G	.	Myocardial infarction	Risk factor
chr17	*AKAP10*	19812541	rs203462	T	C	SNP HET	Missense variant	c.1936A>G	p.Ile646Val	Cardiac conduction defect, susceptibility to	Risk factor

The dot in HGVS.p column stands for no codon change caused by the variant substitution.

**Table 2:** Rare variants associated with cancer genes, of which the allele frequency in the Genome Aggregation Database (GnomAD) is < 0.01.

Chromosome	Gene	Position	Rs ID	Reference	Alternative	Variant Type	Consequence	HGVS.c	HGVS.p
chr2	*MERTK*	112766060	rs112541306	C	T	SNP HET	Splice region variant and intron variant	c.1960+8C>T	.
chr5	*CSF1R*	149456893	rs3829986	C	T	SNP HET	Missense variant	c.835G>A	p.Val279Met
chr5	*SDHA*	256483	rs112307877	CTT	C	DEL HET	Frameshift variant	c.1945	1946delTT
chr6	*MYB*	135518255	rs182817536	C	T	SNP HET	Missense variant	c.1360C>T	p.Arg454Cys
chr6	*ROS1*	117717348	rs79119625	T	C	SNP HET	Splice region variant and intron variant	c.856+3A>G	.
chr8	*PCM1*	17796382	rs754721723	AC	GT	MNP HET	Missense variant	c.476_477delACinsGT	p.Asn159Ser
chr8	*RB1CC1*	53543090	rs770160515	TA	T	DEL HET	Splice region variant and intron variant	c.4441-3delT	.
chr8	*RB1CC1*	53586746	rs77653001	C	T	SNP HET	Missense variant	c.661G>A	p.Asp221Asn
chr9	*PTCH1*	98270531	rs143494325	C	A	SNP HET	Missense variant	c.113G>T	p.Gly38Val
chr10	*DMBT1*	124329672	rs34118835	TC	CT	MNP HOM	Splice region variant and intron variant	c.92-6_92-5delTCinsCT	.
chr10	*FGFR2*	123274705	rs772986332	T	C	SNP HET	Missense variant	c.1216A>G	p.Lys406Glu
chr16	*MYH11*	15808856	rs79129097	T	C	SNP HET	Missense variant	c.5717A>G	p.Asn1906Ser
chr16	*MYH11*	15831299	rs201955317	C	T	SNP HET	Splice region variant and intron variant	c.3314+7G>A	.
chr22	*BCR*	23654017	rs879255379	G	A	SNP HET	Missense variant	c.3316G>A	p.Asp1106Asn
chrX	*BRCC3*	154348267	rs2290069	A	G	SNP HET	Splice region variant and intron variant	c.800-7A>G	.

The dot in HGVS.p column stands for no codon change caused by the variant substitution.

## Data Availability

The data presented in this study are openly available in the NCBI Sequence Read Archive at BioProject accession number PRJNA660915.
